# 3-(2,4-Dibromo­anilino)-2,2-dimethyl-2,3-dihydro­naphtho[1,2-*b*]furan-4,5-dione: a new substituted aryl­amino nor-β-lapachone derivative

**DOI:** 10.1107/S1600536808034545

**Published:** 2008-11-13

**Authors:** Eufrânio N. da Silva, Carlos A. De Simone, Marília O. F. Goulart, Carlos K. Z. Andrade, Raphael S. F. Silva, Antonio V. Pinto

**Affiliations:** a Instituto de Química, Universidade de Brasília, 70910-970 Brasília, DF, Brazil; b Instituto de Química e Biotecnologia, Universidade Federal de Alagoas, 57072-970 Maceió, AL, Brazil; c Nućleo de Pesquisas em Produtos Naturais, Universidade Federal do Rio de Janeiro, 21944-971 Rio de Janeiro, RJ, Brazil

## Abstract

The title compound, C_20_H_15_Br_2_NO_3_, shows the furan ring to adopt a half-chair conformation and the two ring systems to be approximately perpendicular [dihedral angle = 71.0 (2)°]. In the crystal structure, inter­molecular C—H⋯O contacts link the mol­ecules.

## Related literature

For general background, see: Hillard *et al.* (2008 ▶); Pinto *et al.*, (1997 ▶); Dos Santos *et al.* (2001 ▶); Lima *et al.* (2004 ▶). For related structures and biological activity, see: da Silva Júnior *et al.* (2007 ▶, 2008 ▶); Lima *et al.* (2002 ▶). For the synthesis, see: da Silva Júnior *et al.* (2007 ▶, 2008 ▶). For geometric analysis, see: Cremer & Pople (1975 ▶).
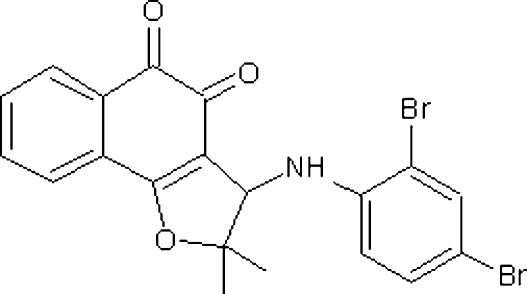


## Experimental

### 

#### Crystal data

C_20_H_15_Br_2_NO_3_
*M* *_r_* = 477.15
Triclinic, 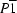

*a* = 8.1430 (3) Å
*b* = 11.2584 (4) Å
*c* = 11.4742 (5) Å
α = 112.073 (2)°
β = 95.546 (2)°
γ = 108.696 (2)°
*V* = 894.70 (6) Å^3^
*Z* = 2
Mo *K*α radiation
μ = 4.55 mm^−1^
*T* = 293 (2) K
0.31 × 0.28 × 0.16 mm

#### Data collection

Nonius KappaCCD diffractometer
Absorption correction: multi-scan (*SADABS*; Sheldrick, 1996 ▶) *T*_min_ = 0.272, *T*_max_ = 0.490
9784 measured reflections
4099 independent reflections
3610 reflections with *I* > 2σ(*I*)
*R*_int_ = 0.032

#### Refinement

*R*[*F* ^2^ > 2σ(*F* ^2^)] = 0.048
*wR*(*F* ^2^) = 0.139
*S* = 1.09
4070 reflections
235 parameters
H-atom parameters constrained
Δρ_max_ = 0.59 e Å^−3^
Δρ_min_ = −1.49 e Å^−3^

### 

Data collection: *COLLECT* (Nonius, 2000 ▶); cell refinement: *SCALEPACK* (Otwinowski & Minor, 1997 ▶); data reduction: *DENZO* (Otwinowski & Minor, 1997 ▶) and *SCALEPACK*; program(s) used to solve structure: *SHELXS97* (Sheldrick, 2008 ▶); program(s) used to refine structure: *SHELXL97* (Sheldrick, 2008 ▶); molecular graphics: *ORTEP-3 for Windows* (Farrugia, 1997 ▶); software used to prepare material for publication: *WinGX* (Farrugia, 1999 ▶).

## Supplementary Material

Crystal structure: contains datablocks I, global. DOI: 10.1107/S1600536808034545/tk2311sup1.cif

Structure factors: contains datablocks I. DOI: 10.1107/S1600536808034545/tk2311Isup2.hkl

Additional supplementary materials:  crystallographic information; 3D view; checkCIF report

## Figures and Tables

**Table 1:** Hydrogen-bond geometry (Å, °)

*D*—H⋯*A*	*D*—H	H⋯*A*	*D*⋯*A*	*D*—H⋯*A*
C3—H3⋯O2^i^	0.98	2.64	3.347 (6)	129
C1′′—H1*A*⋯O2^i^	0.96	2.67	3.389 (6)	132

Symmetry code: (i) 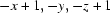.

**Fig. 1.**
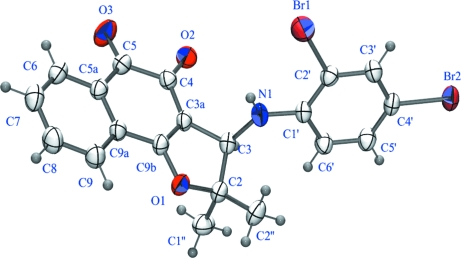
Projection of a molecule of (IV), showing the atom labelling with 50% probability displacement ellipsoids.

## supplementary crystallographic information

### Comment

The search for substances with pharmacological activity has grown exponentially in recent years and quinones play a major role as bioreductive drugs, reactive oxygen species (ROS) enhancers, and redox catalysts (Hillard *et al.*, 2008). Substances such as lapachol (I), (Pinto *et al.*, 1997; dos Santos *et al.*, 2001;  Lima *et al.*, 2004), β-lapachone (II) (Hillard *et al.*, 2008) and nor-β-lapachone (III) (da Silva Júnior *et al.*, 2007), Scheme 1, are prototypes that can be used as starting points for the synthesis of new bioactive molecules. In this context, different compounds with anti-cancer activity (da Silva Júnior *et al.*, 2007), trypanocides (da Silva Júnior *et al.*, 2008), molluscicides (Lima *et al.*, 2002) among others, were synthesized. The introduction of arylamino groups in the furan ring of III retained/enhanced the anticancer activity against six cancer cell lines with IC_50_ values below 1µg/ml (da Silva Júnior *et al.*, 2007), as well as intensified trypanocidal activity (da Silva Júnior *et al.*, 2008), demonstrating clearly that the arylamino group is important for pharmacological activity. In this paper, we report the molecular and crystal structure of (IV) that was easily obtained as described in the literature (da Silva Júnior *et al.*, 2007; 2008).

The atoms of the naphthoquinonic ring of (IV), Fig. 1, are co-planar and the greatest deviation from their least-squares plane is exhibited by atom C5 [0.069 (4) Å]. The O1 atom lies in the mean least-square plane of the naphthoquinonic ring with a deviation of 0.051 (3) Å while atoms O2 and O3 are -0.129 (3) and 0.212 (4)Å out of that plane, respectively. The furane ring adopts a half chair conformation and the calculated puckering parameters are: q_2_ = 0.229 (4) Å and φ = -12 8.04 (1)° (Cremer & Pople, 1975). The dihedral angle between planes passing through atoms C1'-C6' of the aromatic ring and the naphthoquinonic ring is 71.0 (2) °. In the crystal packing, molecules interact through two intermolecular C—H···O contacts, Table 1.

### Experimental

To a chloroform (25mL) solution of the nor-lapachol (2-hydroxy-3-(2-methylprop-1-enyl)naphthalene-1,4-dione, 228 mg, 1 mmol), bromine (2 mL, 38 mmol) was added. The bromo intermediate, 3-bromo-2,2-dimethyl-2,3-dihydro-naphtho[1,2-*b*]furan-4,5-dione, precipitated immediately as an orange solid. Over this mixture, an excess of 2,4-dibromobenzenamine was added and the mixture was left under agitation overnight. After the addition of water (50 ml), the organic phase was separated and washed with 10% HCl (3 *x* 50 ml), dried over sodium sulfate, filtered, and concentrated under reduced pressure. The arylaminoderivative,3-(2',4'-dibromophenylamine)-2H,3*H*-2,2- dimethylnaphtho[1,2 - b]furan-4,5-dione species, was purified by column chromatography over silica-gel, using as eluent a gradient mixture of hexane/ethyl acetate (9/1 to 7/3) with increasing polarity and obtained as a red solid (330 mg, 0.70 mmol, 70% yield). ^1^H NMR (300 MHz, CDCl_3_) δ: 8.14 (1*H*, dd, *J* = 6.7, 1.3 Hz), 7.76–7.63 (3*H*, m), 7.56 (1*H*, d, *J* = 2.0 Hz), 7.30 (1*H*, dd, *J* = 8.8, 2.0 Hz), 6.53 (1*H*, d, *J* = 8.8 Hz), 4.83 (1*H*, d, *J* = 7.5 Hz), 4.48 (NH, d,*J* = 7.5 Hz), 1.66 (3*H*, s), 1.54 (3*H*, s). ^13^C NMR (75 MHz, CDCl_3_) δ: 180.6 (C=O), 175.0 (C=O), 169.7 (C_q_), 143.0 (C_q_), 134.6 (CH), 132.6 (CH),131.1 (CH), 131.0 (C_q_), 129.5 (CH), 127.1 (C_q_), 125.1 (CH), 114.4 (C_q_),112.6(CH), 110.2 (C_q_), 108.9 (C_q_), 96.0 (C_q_), 134.5(CH), 61.2 (CH), 27.5 (CH_3_), 21.6 (CH_3_).

### Refinement

H atoms were located on stereochemical grounds and refined with fixed geometry, each riding on a carrier atom, with N—H = 0.86 Å and C—H = 0.93 - 0.98 Å, and with *U*(H) set to 1.2–1.5 times *U*_eq_(N, C). The maximum and minimum residual electron density peaks were located 0.59 and -1.49 Å, respectively, from the Br1 atom.

### Crystal data

**Table:**

C_20_H_15_Br_2_NO_3_	*Z* = 2
*M**_r_* = 477.15	*F*_000_ = 472
Triclinic, *P*1	*D*_x_ = 1.771 Mg m^−^^3^
Hall symbol: -P 1	Mo *K*α radiation λ = 0.71073 Å
*a* = 8.1430 (3) Å	Cell parameters from 7882 reflections
*b* = 11.2584 (4) Å	θ = 1.0–27.5º
*c* = 11.4742 (5) Å	µ = 4.55 mm^−^^1^
α = 112.073 (2)º	*T* = 293 (2) K
β = 95.546 (2)º	Plate, colorless
γ = 108.696 (2)º	0.31 × 0.28 × 0.16 mm
*V* = 894.70 (6) Å^3^	

### Data collection

**Table:**

Nonius KappaCCD diffractometer	4099 independent reflections
Radiation source: Enraf Nonius FR590	3610 reflections with *I* > 2σ(*I*)
Monochromator: horizonally mounted graphite crystal	*R*_int_ = 0.032
Detector resolution: 9 pixels mm^-1^	θ_max_ = 27.5º
CCD rotation images, thick slices scans	θ_min_ = 2.7º
Absorption correction: multi-scan(SADABS; Sheldrick, 1996)	*h* = −10→9
*T*_min_ = 0.272, *T*_max_ = 0.490	*k* = −14→14
9784 measured reflections	*l* = −14→14

### Refinement

**Table:**

Refinement on *F*^2^	Secondary atom site location: difference Fourier map
Least-squares matrix: full	Hydrogen site location: inferred from neighbouring sites
*R*[*F*^2^ > 2σ(*F*^2^)] = 0.048	H-atom parameters constrained
*wR*(*F*^2^) = 0.139	*w* = 1/[σ^2^(*F*_o_^2^) + (0.0642*P*)^2^ + 1.7472*P*] where *P* = (*F*_o_^2^ + 2*F*_c_^2^)/3
*S* = 1.09	(Δ/σ)_max_ < 0.001
4070 reflections	Δρ_max_ = 0.59 e Å^−^^3^
235 parameters	Δρ_min_ = −1.49 e Å^−^^3^
Primary atom site location: structure-invariant direct methods	Extinction correction: none

### Special details

**Table:**

Geometry. All e.s.d.'s (except the e.s.d. in the dihedral angle between two l.s. planes) are estimated using the full covariance matrix. The cell e.s.d.'s are taken into account individually in the estimation of e.s.d.'s in distances, angles and torsion angles; correlations between e.s.d.'s in cell parameters are only used when they are defined by crystal symmetry. An approximate (isotropic) treatment of cell e.s.d.'s is used for estimating e.s.d.'s involving l.s. planes.
Refinement. Refinement of *F*^2^ against ALL reflections. The weighted *R*-factor *wR* and goodness of fit *S* are based on *F*^2^, conventional *R*-factors *R* are based on *F*, with *F* set to zero for negative *F*^2^. The threshold expression of *F*^2^ > σ(*F*^2^) is used only for calculating *R*-factors(gt) *etc*. and is not relevant to the choice of reflections for refinement. *R*-factors based on *F*^2^ are statistically about twice as large as those based on *F*, and *R*- factors based on ALL data will be even larger.

### Fractional atomic coordinates and isotropic or equivalent isotropic displacement parameters (Å^2^)

**Table:**

	*x*	*y*	*z*	*U*_iso_*/*U*_eq_	
Br1	0.13493 (6)	0.15969 (6)	0.15092 (5)	0.06302 (18)	
Br2	0.61904 (6)	0.13670 (4)	−0.16856 (4)	0.04728 (15)	
O1	0.5871 (4)	0.4358 (3)	0.6646 (3)	0.0405 (6)	
O2	0.2827 (4)	−0.0404 (3)	0.4343 (3)	0.0475 (7)	
O3	0.0912 (5)	−0.0405 (3)	0.6162 (4)	0.0647 (10)	
N1	0.4572 (4)	0.2348 (4)	0.3578 (3)	0.0388 (7)	
H1	0.3585	0.2460	0.3654	0.047*	
C2	0.6940 (5)	0.3902 (4)	0.5704 (4)	0.0376 (8)	
C3	0.5652 (5)	0.2412 (4)	0.4710 (4)	0.0343 (7)	
H3	0.6291	0.1787	0.4452	0.041*	
C3A	0.4399 (5)	0.2026 (4)	0.5504 (3)	0.0335 (7)	
C4	0.3082 (5)	0.0695 (4)	0.5251 (4)	0.0355 (7)	
C5	0.1911 (5)	0.0697 (4)	0.6241 (4)	0.0396 (8)	
C5A	0.2076 (5)	0.2057 (4)	0.7271 (4)	0.0356 (7)	
C6	0.0916 (5)	0.2100 (4)	0.8081 (4)	0.0430 (9)	
H6	0.0005	0.1284	0.7967	0.052*	
C7	0.1114 (6)	0.3356 (5)	0.9059 (4)	0.0487 (10)	
H7	0.0348	0.3379	0.9610	0.058*	
C8	0.2437 (7)	0.4573 (5)	0.9222 (5)	0.0503 (10)	
H8	0.2560	0.5413	0.9882	0.060*	
C9	0.3589 (6)	0.4550 (4)	0.8403 (4)	0.0449 (9)	
H9	0.4480	0.5374	0.8514	0.054*	
C9A	0.3410 (5)	0.3304 (4)	0.7427 (4)	0.0347 (7)	
C9B	0.4547 (5)	0.3204 (4)	0.6524 (4)	0.0339 (7)	
C1'	0.4990 (5)	0.2127 (4)	0.2403 (4)	0.0336 (7)	
C2'	0.3688 (5)	0.1795 (4)	0.1324 (4)	0.0349 (7)	
C3'	0.4010 (5)	0.1562 (4)	0.0115 (4)	0.0384 (8)	
H3'	0.3101	0.1327	−0.0586	0.046*	
C4'	0.5725 (5)	0.1686 (4)	−0.0031 (4)	0.0370 (8)	
C5'	0.7048 (6)	0.2006 (5)	0.1004 (4)	0.0428 (9)	
H5'	0.8189	0.2081	0.0898	0.051*	
C6'	0.6691 (5)	0.2220 (4)	0.2212 (4)	0.0406 (8)	
H6'	0.7597	0.2429	0.2904	0.049*	
C1''	0.8530 (6)	0.3854 (5)	0.6478 (5)	0.0509 (10)	
H1A	0.8108	0.3159	0.6791	0.076*	
H1B	0.9175	0.4744	0.7201	0.076*	
H1C	0.9308	0.3628	0.5930	0.076*	
C2''	0.7497 (6)	0.4983 (4)	0.5182 (5)	0.0466 (9)	
H2A	0.6454	0.4971	0.4703	0.070*	
H2B	0.8268	0.4777	0.4621	0.070*	
H2C	0.8124	0.5886	0.5891	0.070*	

### Atomic displacement parameters (Å^2^)

**Table:**

	*U*^11^	*U*^22^	*U*^33^	*U*^12^	*U*^13^	*U*^23^
Br1	0.0428 (3)	0.0937 (4)	0.0536 (3)	0.0282 (3)	0.0146 (2)	0.0306 (3)
Br2	0.0576 (3)	0.0534 (3)	0.0380 (2)	0.0228 (2)	0.02256 (19)	0.02340 (19)
O1	0.0463 (15)	0.0328 (12)	0.0396 (14)	0.0101 (11)	0.0189 (12)	0.0153 (11)
O2	0.0541 (17)	0.0357 (14)	0.0432 (16)	0.0129 (12)	0.0138 (13)	0.0105 (12)
O3	0.075 (2)	0.0385 (16)	0.074 (2)	0.0084 (15)	0.0367 (19)	0.0246 (16)
N1	0.0419 (17)	0.0533 (19)	0.0326 (16)	0.0262 (15)	0.0153 (13)	0.0222 (14)
C2	0.0393 (19)	0.0401 (19)	0.0381 (19)	0.0139 (15)	0.0151 (16)	0.0217 (16)
C3	0.0386 (18)	0.0405 (18)	0.0338 (18)	0.0199 (15)	0.0151 (15)	0.0209 (15)
C3A	0.0394 (18)	0.0368 (17)	0.0285 (16)	0.0143 (15)	0.0105 (14)	0.0184 (14)
C4	0.0398 (19)	0.0353 (17)	0.0323 (18)	0.0144 (15)	0.0083 (14)	0.0157 (14)
C5	0.0404 (19)	0.0386 (19)	0.041 (2)	0.0121 (15)	0.0120 (16)	0.0207 (16)
C5A	0.0364 (18)	0.0389 (18)	0.0357 (18)	0.0140 (15)	0.0106 (15)	0.0204 (15)
C6	0.0378 (19)	0.050 (2)	0.048 (2)	0.0166 (17)	0.0189 (17)	0.0268 (18)
C7	0.051 (2)	0.062 (3)	0.045 (2)	0.028 (2)	0.0240 (19)	0.027 (2)
C8	0.061 (3)	0.049 (2)	0.042 (2)	0.025 (2)	0.022 (2)	0.0159 (18)
C9	0.054 (2)	0.0382 (19)	0.041 (2)	0.0149 (17)	0.0172 (18)	0.0162 (17)
C9A	0.0393 (18)	0.0355 (17)	0.0302 (17)	0.0124 (14)	0.0101 (14)	0.0165 (14)
C9B	0.0392 (18)	0.0356 (17)	0.0329 (17)	0.0141 (14)	0.0123 (14)	0.0203 (14)
C1'	0.0389 (18)	0.0373 (17)	0.0314 (17)	0.0177 (15)	0.0121 (14)	0.0185 (14)
C2'	0.0329 (17)	0.0388 (18)	0.0382 (19)	0.0162 (14)	0.0122 (14)	0.0192 (15)
C3'	0.043 (2)	0.043 (2)	0.0331 (18)	0.0185 (16)	0.0097 (15)	0.0195 (16)
C4'	0.046 (2)	0.0383 (18)	0.0341 (18)	0.0186 (16)	0.0189 (16)	0.0199 (15)
C5'	0.041 (2)	0.055 (2)	0.044 (2)	0.0241 (18)	0.0193 (17)	0.0271 (19)
C6'	0.0374 (19)	0.055 (2)	0.0371 (19)	0.0220 (17)	0.0111 (15)	0.0235 (17)
C1''	0.042 (2)	0.054 (2)	0.055 (3)	0.0127 (18)	0.0059 (19)	0.029 (2)
C2''	0.052 (2)	0.044 (2)	0.056 (3)	0.0180 (18)	0.027 (2)	0.0305 (19)

### Geometric parameters (Å, °)

**Table:**

Br1—C2'	1.887 (4)	C7—C8	1.377 (7)
Br2—C4'	1.895 (4)	C7—H7	0.9300
O1—C9B	1.344 (4)	C8—C9	1.389 (6)
O1—C2	1.497 (5)	C8—H8	0.9300
O2—C4	1.219 (5)	C9—C9A	1.379 (6)
O3—C5	1.210 (5)	C9—H9	0.9300
N1—C1'	1.374 (5)	C9A—C9B	1.451 (5)
N1—C3	1.461 (5)	C1'—C2'	1.394 (5)
N1—H1	0.8600	C1'—C6'	1.400 (5)
C2—C2''	1.514 (5)	C2'—C3'	1.379 (5)
C2—C1''	1.525 (6)	C3'—C4'	1.392 (5)
C2—C3	1.556 (5)	C3'—H3'	0.9300
C3—C3A	1.501 (5)	C4'—C5'	1.372 (6)
C3—H3	0.9800	C5'—C6'	1.391 (6)
C3A—C9B	1.360 (5)	C5'—H5'	0.9300
C3A—C4	1.436 (5)	C6'—H6'	0.9300
C4—C5	1.552 (5)	C1''—H1A	0.9600
C5—C5A	1.496 (5)	C1''—H1B	0.9600
C5A—C6	1.387 (5)	C1''—H1C	0.9600
C5A—C9A	1.406 (5)	C2''—H2A	0.9600
C6—C7	1.382 (6)	C2''—H2B	0.9600
C6—H6	0.9300	C2''—H2C	0.9600
			
C9B—O1—C2	107.1 (3)	C9A—C9—H9	120.0
C1'—N1—C3	125.9 (3)	C8—C9—H9	120.0
C1'—N1—H1	117.0	C9—C9A—C5A	119.9 (4)
C3—N1—H1	117.0	C9—C9A—C9B	122.9 (3)
O1—C2—C2''	106.1 (3)	C5A—C9A—C9B	117.2 (3)
O1—C2—C1''	106.1 (3)	O1—C9B—C3A	114.0 (3)
C2''—C2—C1''	112.7 (3)	O1—C9B—C9A	119.7 (3)
O1—C2—C3	104.0 (3)	C3A—C9B—C9A	126.2 (3)
C2''—C2—C3	116.4 (3)	N1—C1'—C2'	119.9 (3)
C1''—C2—C3	110.5 (3)	N1—C1'—C6'	123.5 (3)
N1—C3—C3A	106.9 (3)	C2'—C1'—C6'	116.6 (3)
N1—C3—C2	114.7 (3)	C3'—C2'—C1'	123.2 (3)
C3A—C3—C2	100.7 (3)	C3'—C2'—Br1	118.3 (3)
N1—C3—H3	111.3	C1'—C2'—Br1	118.5 (3)
C3A—C3—H3	111.3	C2'—C3'—C4'	118.5 (3)
C2—C3—H3	111.3	C2'—C3'—H3'	120.8
C9B—C3A—C4	121.3 (3)	C4'—C3'—H3'	120.8
C9B—C3A—C3	108.9 (3)	C5'—C4'—C3'	120.3 (3)
C4—C3A—C3	129.6 (3)	C5'—C4'—Br2	120.6 (3)
O2—C4—C3A	125.6 (4)	C3'—C4'—Br2	119.0 (3)
O2—C4—C5	118.8 (3)	C4'—C5'—C6'	120.3 (4)
C3A—C4—C5	115.6 (3)	C4'—C5'—H5'	119.8
O3—C5—C5A	122.3 (4)	C6'—C5'—H5'	119.8
O3—C5—C4	118.7 (4)	C5'—C6'—C1'	121.0 (4)
C5A—C5—C4	119.0 (3)	C5'—C6'—H6'	119.5
C6—C5A—C9A	119.5 (4)	C1'—C6'—H6'	119.5
C6—C5A—C5	120.3 (3)	C2—C1''—H1A	109.5
C9A—C5A—C5	120.3 (3)	C2—C1''—H1B	109.5
C7—C6—C5A	119.9 (4)	H1A—C1''—H1B	109.5
C7—C6—H6	120.0	C2—C1''—H1C	109.5
C5A—C6—H6	120.0	H1A—C1''—H1C	109.5
C8—C7—C6	120.5 (4)	H1B—C1''—H1C	109.5
C8—C7—H7	119.7	C2—C2''—H2A	109.5
C6—C7—H7	119.7	C2—C2''—H2B	109.5
C7—C8—C9	120.2 (4)	H2A—C2''—H2B	109.5
C7—C8—H8	119.9	C2—C2''—H2C	109.5
C9—C8—H8	119.9	H2A—C2''—H2C	109.5
C9A—C9—C8	119.9 (4)	H2B—C2''—H2C	109.5
			
C9B—O1—C2—C2''	143.2 (3)	C8—C9—C9A—C5A	−0.8 (6)
C9B—O1—C2—C1''	−96.7 (3)	C8—C9—C9A—C9B	179.1 (4)
C9B—O1—C2—C3	19.9 (4)	C6—C5A—C9A—C9	2.0 (6)
C1'—N1—C3—C3A	−154.9 (4)	C5—C5A—C9A—C9	−178.1 (4)
C1'—N1—C3—C2	94.4 (4)	C6—C5A—C9A—C9B	−177.9 (4)
O1—C2—C3—N1	92.0 (3)	C5—C5A—C9A—C9B	2.1 (5)
C2''—C2—C3—N1	−24.3 (5)	C2—O1—C9B—C3A	−8.8 (4)
C1''—C2—C3—N1	−154.5 (3)	C2—O1—C9B—C9A	172.6 (3)
O1—C2—C3—C3A	−22.4 (3)	C4—C3A—C9B—O1	178.5 (3)
C2''—C2—C3—C3A	−138.7 (3)	C3—C3A—C9B—O1	−6.9 (4)
C1''—C2—C3—C3A	91.1 (4)	C4—C3A—C9B—C9A	−3.0 (6)
N1—C3—C3A—C9B	−101.7 (4)	C3—C3A—C9B—C9A	171.6 (3)
C2—C3—C3A—C9B	18.4 (4)	C9—C9A—C9B—O1	1.7 (6)
N1—C3—C3A—C4	72.3 (5)	C5A—C9A—C9B—O1	−178.5 (3)
C2—C3—C3A—C4	−167.6 (4)	C9—C9A—C9B—C3A	−176.7 (4)
C9B—C3A—C4—O2	178.0 (4)	C5A—C9A—C9B—C3A	3.1 (6)
C3—C3A—C4—O2	4.7 (7)	C3—N1—C1'—C2'	166.3 (3)
C9B—C3A—C4—C5	−2.1 (5)	C3—N1—C1'—C6'	−14.0 (6)
C3—C3A—C4—C5	−175.4 (3)	N1—C1'—C2'—C3'	179.8 (3)
O2—C4—C5—O3	7.7 (6)	C6'—C1'—C2'—C3'	0.1 (5)
C3A—C4—C5—O3	−172.1 (4)	N1—C1'—C2'—Br1	−2.3 (5)
O2—C4—C5—C5A	−173.3 (4)	C6'—C1'—C2'—Br1	178.0 (3)
C3A—C4—C5—C5A	6.8 (5)	C1'—C2'—C3'—C4'	−1.1 (6)
O3—C5—C5A—C6	−8.0 (6)	Br1—C2'—C3'—C4'	−179.0 (3)
C4—C5—C5A—C6	173.1 (4)	C2'—C3'—C4'—C5'	1.3 (6)
O3—C5—C5A—C9A	172.0 (4)	C2'—C3'—C4'—Br2	179.9 (3)
C4—C5—C5A—C9A	−6.9 (5)	C3'—C4'—C5'—C6'	−0.4 (6)
C9A—C5A—C6—C7	−2.1 (6)	Br2—C4'—C5'—C6'	−179.1 (3)
C5—C5A—C6—C7	177.9 (4)	C4'—C5'—C6'—C1'	−0.6 (6)
C5A—C6—C7—C8	1.2 (7)	N1—C1'—C6'—C5'	−179.0 (4)
C6—C7—C8—C9	0.0 (7)	C2'—C1'—C6'—C5'	0.7 (6)
C7—C8—C9—C9A	−0.1 (7)		

### Hydrogen-bond geometry (Å, °)

**Table:**

*D*—H···*A*	*D*—H	H···*A*	*D*···*A*	*D*—H···*A*
C3—H3···O2^i^	0.98	2.64	3.347 (6)	129
C1''—H1A···O2^i^	0.96	2.67	3.389 (6)	132

Symmetry codes: (i) −*x*+1, −*y*, −*z*+1.
